# Bio-Inspired Photoelectric Dual-Mode Sensor Based on Photonic Crystals for Human Motion Sensing and Monitoring

**DOI:** 10.3390/gels10080506

**Published:** 2024-08-01

**Authors:** Wenxiang Zheng, Zhibin Wang, Mengnan Zhang, Yanxin Niu, Yuchuan Wu, Pengxin Guo, Niu Zhang, Zihui Meng, Ghulam Murtaza, Lili Qiu

**Affiliations:** 1 School of Chemistry and Chemical Engineering, Beijing Institute of Technology, Beijing 100081, China; zhengwenxiang2019@163.com (W.Z.); 3220231727@bit.edu.cn (Z.W.); 3120231227@bit.edu.cn (M.Z.); 3120231223@bit.edu.cn (Y.N.); 3120221235@bit.edu.cn (Y.W.); mengzh@bit.edu.cn (Z.M.); 2 Analysis & Testing Centre, Beijing Institute of Technology, Beijing 100081, China; niuzhang2019@bit.edu.cn; 3 School of Science, Minzu University of China, Beijing 100074, China

**Keywords:** photonic crystals, photoelectric, dual-mode signal, sensing and monitoring, joint motion

## Abstract

Photoelectric dual-mode sensors, which respond to strain signal through photoelectric dual-signals, hold great promise as wearable sensors in human motion monitoring. In this work, a photoelectric dual-mode sensor based on photonic crystals hydrogel was developed for human joint motion detection. The optical signal of the sensor originated from the structural color of photonic crystals, which was achieved by tuning the polymethyl methacrylate (PMMA) microspheres diameter. The reflective peak of the sensor, based on 250 nm PMMA PCs, shifted from 623 nm to 492 nm with 100% strain. Graphene was employed to enhance the electrical signal of the sensor, resulting in a conductivity increase from 9.33 × 10^−4^ S/m to 2 × 10^−3^ S/m with an increase in graphene from 0 to 8 mg·mL^−1^. Concurrently, the resistance of the hydrogel with 8 mg·mL^−1^ graphene increased from 160 kΩ to 485 kΩ with a gauge factor (GF) = 0.02 under 100% strain, while maintaining a good cyclic stability. The results of the sensing and monitoring of finger joint bending revealed a significant shift in the reflective peak of the photoelectric dual-mode sensor from 624 nm to 526 nm. Additionally, its resistance change rate was measured at 1.72 with a 90° bending angle. These findings suggest that the photoelectric dual-mode sensor had the capability to detect the strain signal with photoelectric dual-mode signals, and indicates its great potential for the sensing and monitoring of joint motion.

## 1. Introduction

In nature, the bright colors in many organisms stem from the periodic surface microstructure rather than pigments, which are known as structural colors [1,2,3]. Among them, the head feathers of the Anna’s Hummingbird, known as the “nine-headed bird”, use a microstructure that achieves light reflection, refraction, and interference to display changing colors at different viewing angles [4]. Inspired by the microstructure of organisms, photonic crystals (PCs) are artificial periodic microstructures that function similar to semiconductors [5,6,7], whose photonic bandgap (PBG) is formed from the light diffraction of their periodic structures [8,9,10]. Similar to electronic bandgaps in semiconductors, wavelengths within the PBGs of PCs are fully reflected and cannot propagate. When PBG falls within the visible light range, PCs exhibit a structural color, which is their most important optical feature. Artificial PCs with a specific PBG can be designed by tuning the effective refractive index and lattice distance of the materials [11]. Because of their unique optical properties, a series of photonic crystals composites were created by combining PCs with functional materials, and they have attracted extensive attention. Their specific responses to external stimuli have caused them to be highly effective in the fields of physical, chemical, and biological visualization [12,13,14,15].

Wearables are devices that integrate bio-compatible composite materials into clothing, accessories, or directly onto the skin surface for monitoring human joint motion, body temperature, bodily secretions, and environmental gases [16,17,18,19,20]. NIPAM-based hydrogels have attracted significant attention in wearable optical sensing due to their temperature-sensitive properties, which can shrink their volume as the temperature increases [21,22]. Multifunctional NIPAM-based hydrogels can be obtained by introducing acrylamide (AM), poly-pyrrole (PPy), and silk fibroin to satisfy different applications [23,24,25,26]. Zhang et al. [27] fabricated an interactive sensor based on PCs with flexible materials, resulting in a dynamically visual response of structural color for real-time monitoring of joint movement in the human body. Xue et al. [28] developed a structure–color hydrogel with inverse opal PCs that enabled the dual-mode signal output of electrons and structural colors. This hydrogel was applied to provide quantitative feedback for external stimuli such as stretching, bending, compression, and temperature. Although a series of hydrogel sensors based on PCs have been successfully developed and matured [29,30], the inherent volatility of water and weak mechanical properties of traditional hydrogels limit their practical application. Therefore, it is necessary to overcome the limitations of hydrogels, and combine them with PCs to develop a visualization sensor with a stable signal output.

In this work, a photoelectric dual-mode sensor with PCs and NIPAM-AM hydrogel doped with graphene was developed for the detection of human joint motion. The optical properties of the sensor were optimized by tuning the diameters of the polymethyl methacrylate (PMMA) microspheres. The reflective peaks of the photoelectric dual-mode sensor based on 250 nm PMMA PCs exhibited a blue shift in response to tensile strain and increasing temperature. The introduced graphene enhanced the conductivity of the sensor, while the glycerol optimized the volatile water and strengthened the weak mechanical properties of the NIPAM-AM hydrogel. The results from sensing and monitoring the bending of finger joints indicated that the photoelectric dual-mode sensor could detect the strain signal in the form of photoelectric dual-mode signals. This capability holds great potential for sensing and monitoring joint motion.

## 2. Results and Discussion

### 2.1. PMMA Microspheres and PMMA Photonic Crystals

**Figure 1:**
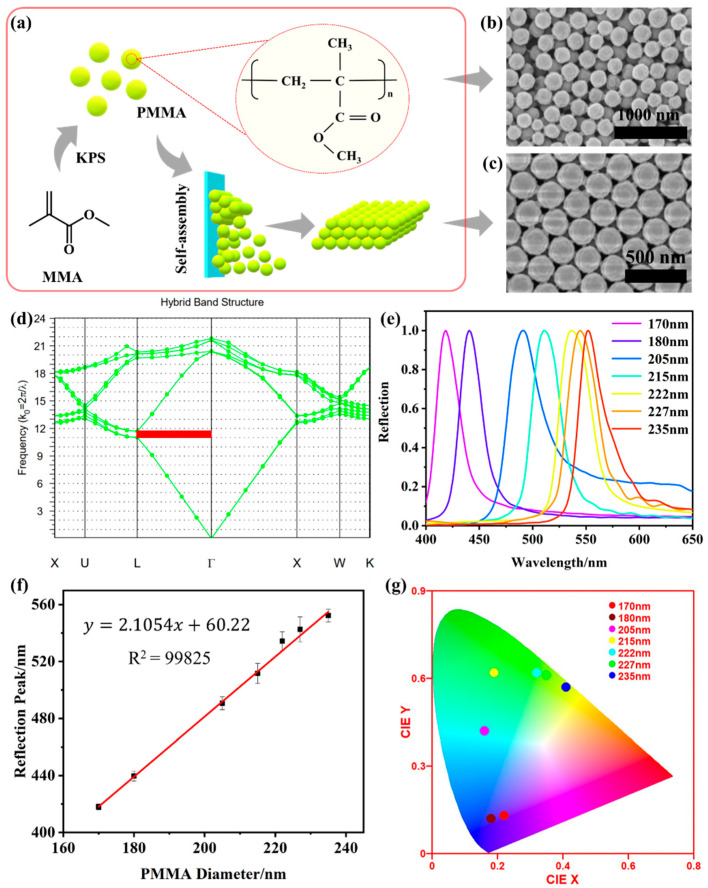
(**a**) Preparation of PMMA microspheres and PMMA photonic crystals; the SEM of (**b**) 235 nm PMMA microspheres and (**c**) 235 nm PMMA photonic crystals; (**d**) the simulated result of 235 nm PMMA photonic crystals with RSOFT software; the PMMA photonic crystals with different diameters; (**e**) reflective spectrum; and (**f**) liner relationship between reflective peaks and PMMA diameter and (**g**) CIE picture.

Figure 1a shows a schematic diagram of the PMMA microspheres obtained with free radical polymerization of methyl methacrylate (MMA), which was polymerized with the induction of thermal initiator potassium persulfate (KPS), and the microspheres were self-assembled into periodic ordered PCs. Figure 1b and Figure S1 are SEM images of a series of PMMA microspheres with different diameters prepared by adjusting the amount of MMA monomer in the reaction system. The diameter of microspheres was correlated with the MMA monomer content, which is the result of more free radicals participating in the polymerization. The obtained PMMA microspheres had a uniform size and good sphericity, which was conducive to self-assembly into a high-ordered face centered cubic (FCC) PCs structure. Figure 1c and Figure S2 display the SEM images of PMMA PCs featuring different diameters. The ordered periodic structures of the microspheres determined their unique optical properties—photonic band gap (PBG). This PBG enables the complete reflection of light waves at specific wavelengths while allowing others to transmit through. PBG prediction is critical for the design of PCs with desired optical properties. In this study, the simulated PBG results of FCC PCs employing PMMA microspheres are shown in Figure 1d and Figure S3. According to the simulated results with the band-solve of RSOFT 2019 software in Figure 1d, the k_0_ of 235 nm PMMA PCs was 11.01~11.688, which can be converted into PBG range of 538 nm~571 nm using the formula of λ = 2π/k_0_. Figure S4 shows that the simulated PBG range increased with the larger PMMA diameter, and the relationship between the simulated reflection peaks and PMMA diameters satisfied y = 2.12714 x + 56.14 with R^2^ = 0.99522, which was in great agreement with the actual reflection peaks in Figure 1e, and the actual reflection peaks and diameters satisfied y = 2.1054 x + 60.22, R^2^ = 0.99825 (Figure 1e). In terms of naked-eye observation, PBG can be macroscopically presented as a vivid structural color, as shown in Figure 1g. The structural colors of PMMA PCs shifted from blue to cyan, green, and yellow when the diameters increased from 170 nm to 235 nm. The above results showed the significant impact of the microsphere diameter on the optical properties of the PMMA PCs, and suggests that simulation can predict the PMMA diameter necessary for achieving specific optical properties in PCs. This prediction can further be applied to guide the manufacturing of PCs for specific fields.

### 2.2. Flexible NIPAM–AM Hydrogel with Graphene

**Figure 2:**
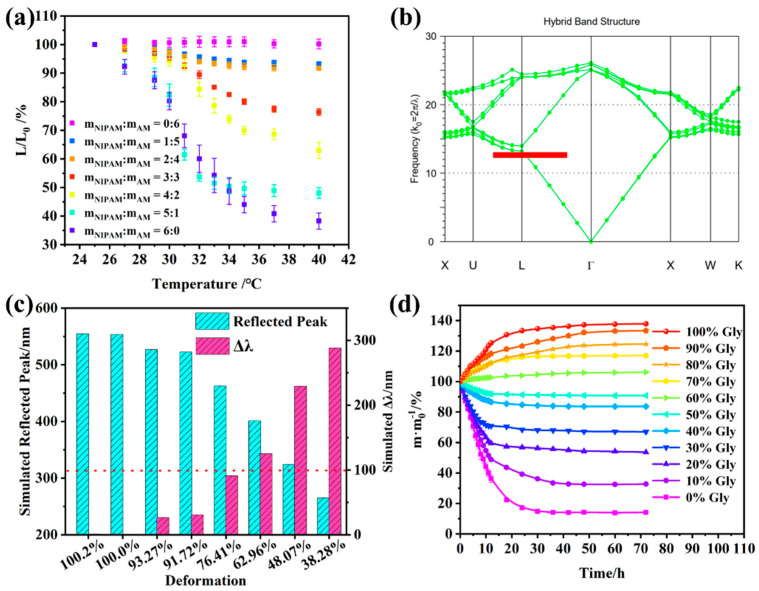
(**a**) The length of NIPAM–AM hydrogels with different AM and NIPAM monomer ratios varied with temperatures; (**b**) simulation results of PBG tuning of 235 nm PMMA PCs with a lattice distance reduced to 76.41% of the initial lattice distance; (**c**) simulation results of reflection peak and Δλ with different lattice distances of 235 nm PMMA PCs; NIPAM–AM hydrogels with m_NIPAM_:m_AM_ = 4:2 immersed in glycerol solution of different concentrations for 48 h, and the immersion solution changed every 12 h; (**d**) the water loss curve at 50% humidity and 35 °C.

The lower critical dissolution temperature (LCST) derived from hydrophilic amide groups and hydrophobic isopropyl groups makes N-Isopropyl acrylamide (NIPAM) suitable for application in temperature-stimulated hydrogels [31,32,33]. Figure 2a shows the volume–temperature curve of NIPAM–AM hydrogels with different proportions of NIPAM and acrylamide (AM), which indicated that the degree of volume shrinkage of the NIPAM–AM hydrogels was positively correlated with NIPAM ratio. And the volume of the NIPAM–AM hydrogel with m NIPAM: m AM = 1:1 shrunk to 76.41%, which would effectively reduce the lattice distance of the PCs. Using 235 nm PMMA PCs as an illustration (Figure 2b,c and Figure S5), the simulated reflective peak measured 462.6 nm when the lattice distance decreased to 76.41%. This resulted in a blue shift of the reflective peak by 91 nm compared with the initial 553.6 nm. These findings suggest that the approach is viable for temperature-sensitive visualization of NIPAM–AM hydrogels with PMMA PCs.

**Figure 3:**
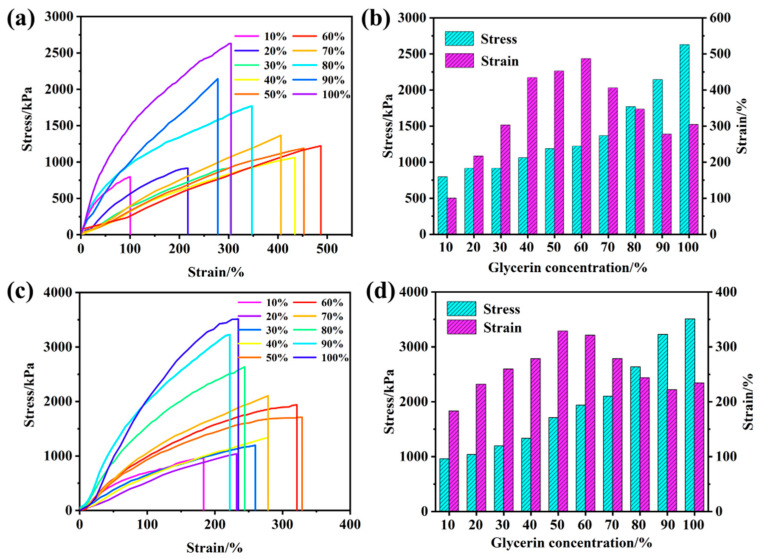
NIPAM–AM hydrogels with m_NIPAM_:m_AM_ = 4:2 immersed in glycerol solutions of different concentrations for 48 h, and the immersion solution changed every 12 h: (**a**) tensile strain curve and (**b**) stress and strain analysis with different glycerol concentrations, (**c**) tensile strain curve after freezing at −23 °C for 24 h, and (**d**) stress and strain analysis with different glycerol concentrations.

Glycerol can form hydrogen bonds with the NIPAM–AM hydrogel network, which is conducive to optimizing their water retention and frost resistance, and improving the mechanical strength of the NIPAM–AM hydrogels. As shown in Figure 2d, the water loss characteristic of the NIPAM–AM hydrogels immersed in different glycerol concentrations system for solvent displacement. The results indicated that the hydrogels without solvent replacement lost their elasticity and became hard at 50% humidity and 35 °C for 72 h, and only 14.13% of initial quality was retained. The water retention performance of the NIPAM–AM hydrogels with solvent replacement was improved (Figure S6), in which the hydrogels mass with 60% glycerol increased slightly after 72 h, and the mass of the NIPAM–AM hydrogels with 100% glycerol became 137.91%, which was the result of high glycerol content and water absorption. The tensile stress-strain results shown in Figure 3a,b are utilized to evaluate the mechanical improvement of NIPAM–AM hydrogels by glycerol. It was observed that the tensile strain initially strengthened and then decreased with the increase in glycerol concentration. The maximum tensile strain reached 487% when the hydrogels were soaked in a 60% glycerol solution. As the glycerol content increased, more hydrogen bonds formed, resulting in reduced mechanical flexibility and increased fracture stress. Specifically, when the NIPAM–AM hydrogel was immersed in a 100% glycerol system, the tensile strain and stress reached 304.8% and 2629 kPa respectively. To explore their frost resistance, the NIPAM–AM hydrogels were cooled at −20 °C for 24 h, the tensile stress—strain test was performed again and the results are shown in Figure 3c,d, compared with the pre-freezing hydrogels, their tensile strain decreased and tensile stress increased, which was the result of hydrogen bonds number increasing during the freezing treatment. Based on the above results, the NIPAM–AM hydrogels with 60% glycerol had excellent water retention, frost resistance and mechanical properties, which can be utilized in wearable flexible sensors for strain and temperature monitoring.

**Figure 4:**
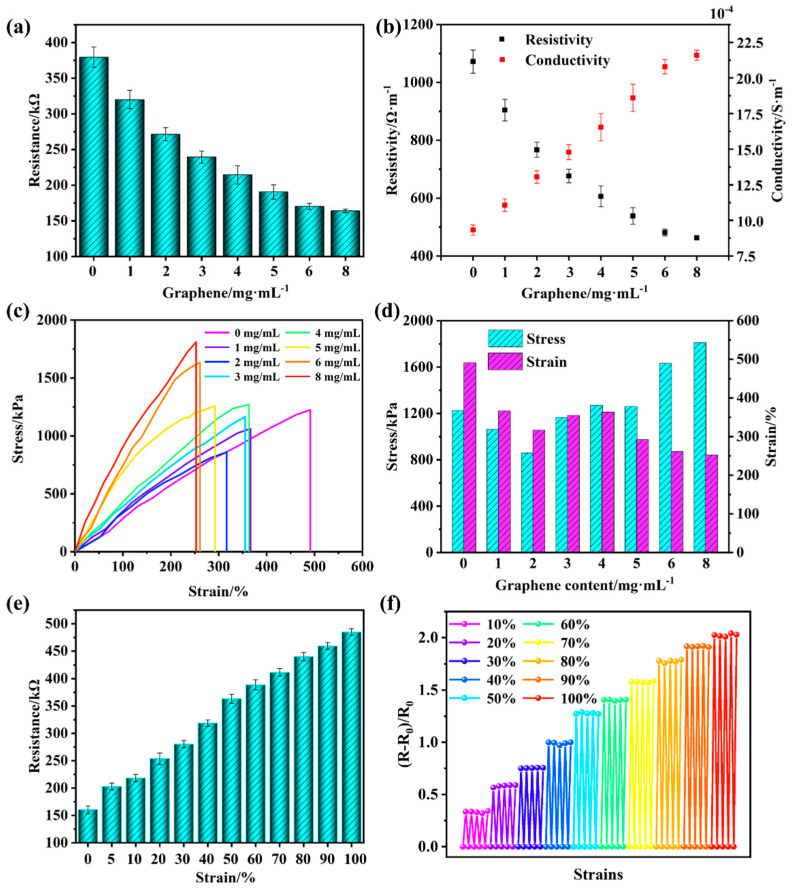
The NIPAM−AM hydrogels with different graphene contents (length of 1 cm, diameter 6 of mm) (**a**) resistance, (**b**) resistivity and conductivity, and (**c**) tensile stress–strain curve; (**d**) stress−strain values; (**e**) the relationship between the resistance of hydrogels with 8 mg·mL^−1^ graphene and strain; and (**f**) the cycling resistance with different strains.

Based on excellent conductive properties [34,35,36], graphene was utilized to enhance the conductivity of the NIPAM–AM hydrogels. As shown in the Figure 4a, the resistance of the NIPAM–AM hydrogel without graphene was 379 kΩ. This resistance decreased from 320 kΩ to 163 kΩ with the increase of graphene content from 1 mg·mL^−1^ to 6 mg·mL^−1^. Additionally, a minor effect on resistance was observed 8 mg·mL^−1^ of graphene. With the increased graphene content, the resistivity of the NIPAM–AM hydrogel was reduced from 1072.17 Ω·m to 463.08 Ω·m and the conductivity increased from 9.33 × 10^−4^ S/m to 2 × 10^−3^ S/m (Figure 4b), which indicated the improvement of the hydrogel conductivity. The mechanical properties of hydrogels weakened with the flake graphene (Figure 4c,d), and the tensile strain of the hydrogel with 8 mg·mL^−1^ was still as high as 253%, which maintained the mechanical flexibility of the NIPAM–AM hydrogel. Figure 4e shows the resistance of the NIPAM–AM hydrogel with 8 mg·mL^−1^ graphene increased significantly from 160 kΩ to 485 kΩ with larger tensile strain, the gauge factor (GF) was 0.02 and had good cyclic stability (Figure 4f), which indicated that the NIPAM–AM hydrogel with graphene can derive strain signal in the form of electrical signal, and can be utilized in the sensor with photoelectric two-mode signals.

### 2.3. The Photoelectric Dual-Mode Sensor

**Figure 5:**
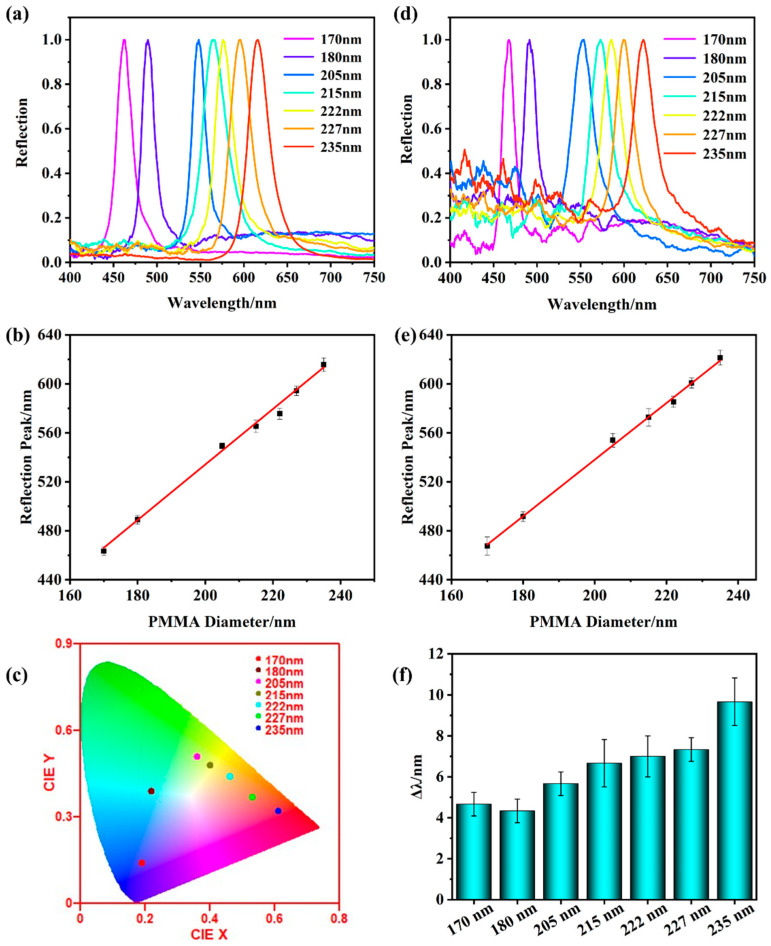
(**a**) The reflective spectrum of the photoelectric dual-mode sensors with different PMMA diameters, (**b**) the relationship between PMMA diameters and reflective peaks and (**c**) the CIE picture; (**d**) the reflective spectrum of the photoelectric dual-mode sensors with 60% glycerol and (**e**) the liner relationship between PMMA diameters and reflective peaks; (**f**) the effect of glycerol on reflective peaks.

The reflective spectrum of photoelectric dual-mode sensor based on PMMA PCs with different diameters and NIPAM–AM hydrogel with graphene is shown in Figure 5a. Their reflective peaks red-shifted from 463 nm to 616 nm with the increase of PMMA diameters from 170 nm to 235 nm. Compared with the reflective peaks of PCs, the reflective peaks were red-shifted by 45 nm~63 nm (Figure S7), attributed to the modification in the effective refractive index and lattice distance due to the substitution of air with NIPAM–AM hydrogel. The SEM of the photoelectric dual-mode sensor shows that the air between PMMA microspheres had been replaced by the NIPAM–AM hydrogel with graphene, and the distance between PMMA microspheres increased (Figure S8), which was consistent with the red shift of the reflective peaks mentioned above. The reflective peaks of the photoelectric dual-mode sensors had a great linear relationship with PMMA diameters, which satisfied y = 2.267x + 80.68 with R^2^ = 0.99245 and could be utilized to guide the preparation of the sensors based on PMMA PCs with specific optical properties (Figure 5b). The macroscopic display of reflection peaks was structural colors, and the structural colors were from blue to cyan, green, yellow, orange, and red when the reflection peak shifted from 463 nm red to 616 nm, which covered the full visible range (Figure 5c). Figure 5d shows the reflective spectrum of the photoelectric dual-mode sensors after immersing in 60% glycerol solution, where their great linear relationship between the reflective peaks and PMMA diameters was maintained and satisfied y = 2.312x + 75.69 with R^2^ = 0.99747 (Figure 5e). The reflective peaks slightly red-shifted when glycerol replaced water in the photoelectric dual-mode sensors (Figure 5f), which was the result of a greater refractive index of glycerol than that of water. Based on the blue-shift of the reflective peaks, caused by the reduction in the PC lattice distance, the photoelectric dual-mode sensor based on 235 nm PMMA PCs was applied to the visual sensing of the temperature and strain in the full visible range.

**Figure 6:**
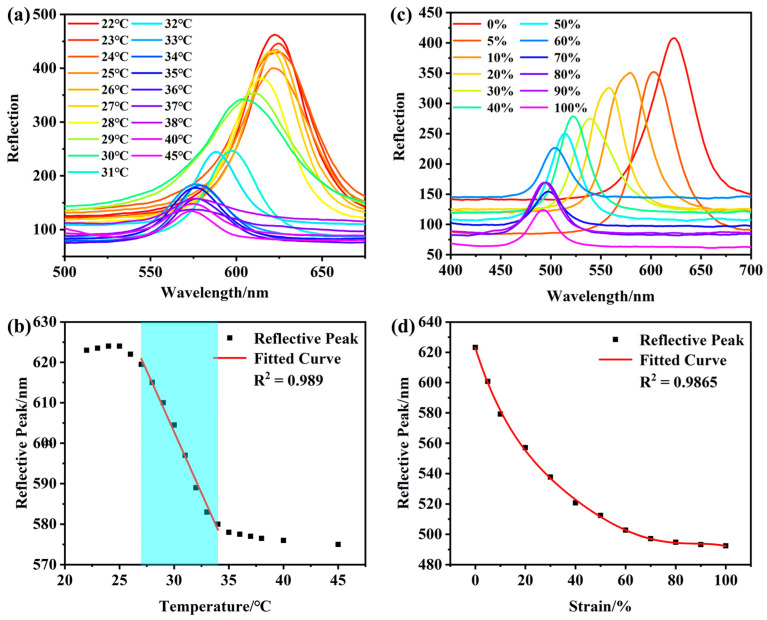
Photoelectric dual-mode sensor based on 235 nm PMMA PCs with different temperatures: (**a**) reflective spectrum; (**b**) the liner relationship between reflective peaks and tensile strain; the photoelectric dual-mode sensor based on 235 nm PMMA PCs with different tensile strain (**c**) reflective spectrum and (**d**) the liner relationship between reflective peaks and tensile strain.

**Figure 7:**
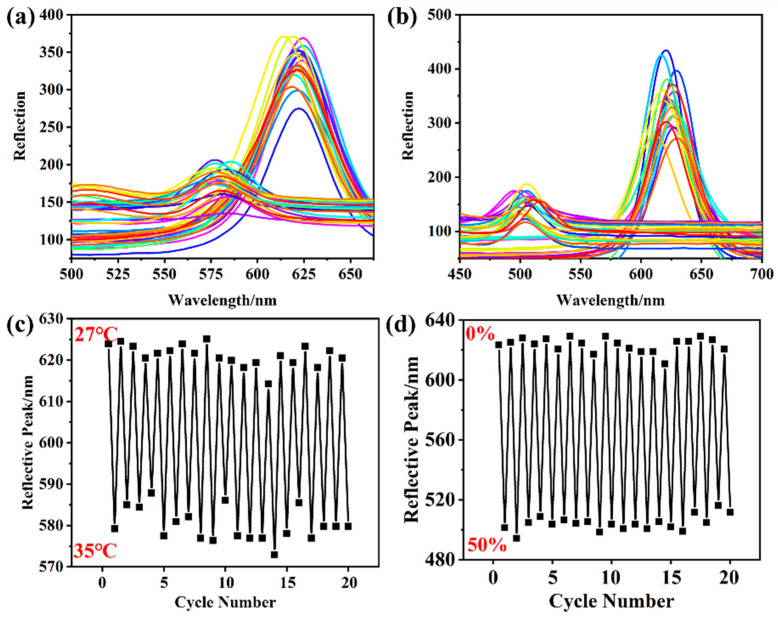
The photoelectric dual-mode sensor based on 235 nm PMMA PCs: (**a**,**b**) temperature cycling characteristics and (**c**,**d**) tensile strain cycling characteristics.

Figure 6a shows the reflective spectrum of the photoelectric dual-mode sensor utilizing 235 nm PMMA PCs at different temperatures. In addition, the NIPAM–AM hydrogel with graphene experienced slight swelling as the temperature rose from 22 °C to 25 °C. This swelling caused an increase in distance between PMMA microspheres and a slight red shift of the reflection peak. The temperature sensitivity of the photoelectric dual-mode sensor was triggered when the temperature further increased from 25 °C to 35 °C, and the shrinkage of the NIPAM–AM hydrogel with graphene lead to a decrease in the lattice distance, which shifted the reflection peak from 622 nm to 577 nm. And a 3 nm shift in the reflective peak with 45 °C indicated that the thermal response range of the sensor was exceeded. Based on the above analysis, the temperature responsive range of the photoelectric dual-mode sensor was estimated to be from 27 °C to 35 °C. Further, a good linear relationship existed between the reflection peaks and temperature, as demonstrated by equation, y = −6.036x + 783.8 with R^2^ = 0.989 (Figure 6b). Figure 7a,b shows that the photoelectric dual-mode sensor had a good cycling stability with RSD = 7.24% of the reflective peak shift. Further, the lattice distance of PMMA microspheres also decreased and caused a shift in the reflective peak when the NIPAM–AM hydrogel with graphene was tensile. As shown in Figure 6c, the reflective peak of the sensor shifted from 623 nm to 492 nm, and the structural color gradually changed from red to yellow, green, cyan, and even blue (Figure S9). The reflection peaks had a nonlinear relationship with the tensile strain with R^2^ = 0.9865 (Figure 6d), and the NIPAM–AM hydrogel with graphene maintained excellent stability with RSD = 5.29% when the hydrogel was tensile by 60% (Figure 7c,d). The results of the reflective peaks shift with temperature and strain stimuli show that the NIPAM–AM hydrogel with graphene could be utilized for the visual optical sensing of temperature and strain.

**Figure 8:**
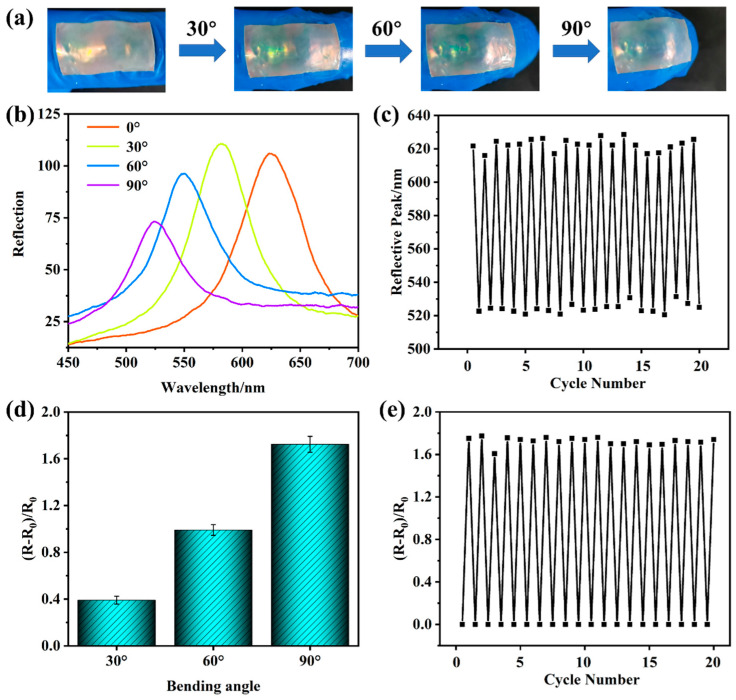
The photoelectric dual-mode sensor based on 235 nm PMMA PCs attached on the finger joint with different bending angles: (**a**) actual pictures, (**b**) reflective spectrum and (**c**) cycling tests, and (**d**) resistance and (**e**) cycling tests when the bending angle ranges from 0° to 90°.

As shown in Figure 8, the photoelectric dual-mode sensor based on 235 nm PMMA PCs was fixed on the surface of a rubber glove for photoelectric dual-mode sensing of joint movement. Figure 8a is the actual pictures of a finger with different bending angles, and the structural color of the photoelectric dual-mode sensor changed from red to green when the joint was bent from 0° to 90°. At the same time, the reflective peak in Figure 8b shifted from 624 nm to 583 nm, 549 nm, and 526 nm sequentially with finger bending from 0° to 30°, 60°, and 90°, and the reflection peak tuning had good cyclic stability in cyclic bending (Figure 8c and Figure S10). The resistance increased from 162 kΩ to 225 kΩ, 322 kΩ, and 440 kΩ as the finger was bent from 0° to 30°, 60°, and 90° (Figure S11), and the corresponding (R − R_0_)/R_0_ was 0.39, 0.99, and 1.72 (Figure 8d), which was great cyclic stability when the finger was bent from 0° to 90° (Figure 8e). Compared to existing work (Table 1), the sensor had a wide spectral response range, excellent stretchability, freeze and water retention characteristics, cycling stability, and a sensitive electrical signal, which is essential for the stable output of sensing signals (Table 1). The above results demonstrate that the photoelectric dual-mode sensor transmitted joint strain signals in the form of optical and electrical signals, which can be utilized in the field of joint movement and health monitoring.

## 3. Conclusions

In summary, a photoelectric dual-mode sensor based on photonic crystals and NIPAM-AM hydrogel doped with graphene was developed for human joint motion detection. The structural colors of the PMMA PCs shifted from blue to cyan, green, and yellow when the diameters increased from 170 nm to 235 nm. Graphene was introduced for enhancing the electrical signal of the sensor, and the conductivity increased from 9.33 × 10^−4^ S/m to 2 × 10^−3^ S/m when the graphene content increased from 0 to 8 mg·mL^−1^. The resistance of the hydrogel with 8 mg·mL^−1^ graphene increased from 160 kΩ to 485 kΩ with 100% strain. Glycerol was utilized to optimize volatile water and strengthen the mechanical properties of the NIPAM-AM hydrogel. The reflective peak of the photoelectric dual-mode sensor based on 250 nm PMMA PCs shifted from 623 nm to 492 nm and the structural color changed from red to green. Monitoring of the finger joint bending results indicated that the reflective peak of the sensor shifted from 624 nm to 526 nm, which shows the photoelectric dual-mode sensor can detect a strain signal in the form of photoelectric dual-mode signals. The above results show that the photoelectric sensor achieved dual-mode signals for the transmission of joint motion, which is expected to emphasize the potential applications in various fields such as healthcare monitoring, sports science, and robotics.

## 4. Materials and Methods

### 4.1. Materials and Reagents

Methyl methacrylate (MMA, A.R.), potassium persulfate (KPS, A.R.), N,N′-methylenebis-acrylamide (BIS, A.R.), ammonium persulfate (APS, A.R.), glycerol (A.R.), and polyethylene glycol (PEG, *Mw* = 20,000) were purchased from J&K. Alumina (Al_2_O_3_, A.R.), acrylamide (AM, A.R.), graphene, and N-isopropylacrylamide (NIPAM, A.R.) were purchased from TCI. Ethylenediaminetetraacetic acid (EDTA, A.R.) and sodium carbonate (Na_2_CO_3_, A.R.) were purchased from SINOPHARM, and the Asian carp fish scales purchased from a local market (Zaozhuang, Shandong, China).

ZEISS Gemini SEM was used to characterize the microstructure of the photonic mate-rials, an optical fiber spectrometer (Avaspec-2048TEC, Avantes, Beijing, China) was used for the optical performance testing, the universal testing machine (AGS-J) was used for the tensile strain test, and the LCR digital bridge (TH2827) was used for electrical signal acquisition.

### 4.2. The Preparation of PMMA Microspheres

PMMA microspheres were prepared by soap-free emulsion polymerization [45]. DI water (150 mL) was added to a 250 mL four-mouth flask and heated to 75 °C. MMA (4 mL, 4.5 mL, 5 mL, 5.5 mL, 6 mL, 6.5 mL, or 7 mL) was added to the reaction system, and the reaction system was stirred at 300 rpm and heated to 80 °C within 15 min, then 5 mL KPS aqueous solution (0.02 g/mL) was added. The reaction system was heated at 80 °C for 45 min, followed by washing with DI water three times, and the PMMA suspensions were obtained. The PMMA microspheres with different diameters were prepared by adjusting the amount of MMA monomer.

### 4.3. Preparation of PMMA Photonic Crystals

The vertical self-assembly method was used to prepare three-dimensional (3D) PCs. In brief, 200 mL 0.2 wt % PMMA solution was prepared and placed in a sample cell, and the hanging slides in the sample cell were hydrophilic with a plasma cleaning instrument. The sample cell containing PMMA solution was placed at 35 °C and 50% humidity until the solvent evaporated completely, and the PMMA microspheres were arranged orderly on the surface of glass slides, which was the photonic crystals.

### 4.4. Preparation of Fish Collagen

Fish collagen was obtained from scales of Asian carp with the hydrothermal method. The scales were washed three times at room temperature (25 °C) with magnetic stirring for 30 min in H_2_O and 1 L 5 g/L Na_2_CO_3_ solution, respectively. Then, the scales were added to 1 L 0.5 g/L EDTA solution and stirred, repeated thrice, and washed thrice with H_2_O. Finally, the scales were added into an 80 °C aqueous with 300 rpm for 60 min to obtain the fish collagen solution. The original fish collagen solution was placed in 8000~14,000 dialysis bags, concentrated in 25 wt % polyethylene glycol solution at 25 °C and stored at −20 °C.

### 4.5. Preparation of NIPAM-AM Hydrogel with Graphene

The NIPAM-AM hydrogel with graphene was prepared using the one pot method. Firstly, 3 g AM, 3 g NIPAM, 0.06 g BIS, 0.1 g APS, and 6 g fish collagen were dissolved in 20 mL H_2_O, which was the A solution. Then, 0.030 g graphene was dispersed evenly in 10 mL 50% of glycerol solution, which was the B solution. The B solution was added to the A solution with magnetic stirring to obtain the hydrogel prepolymer. Finally, the prepolymer was thermally polymerized for 20 min to obtain the NIPAM-AM hydrogel with graphene.

### 4.6. Preparation of Photoelectric Dual-Mode Sensor

The prepared PMMA photonic crystals slide was fixed onto a glass plate, 10 layers tape was affixed at both ends, and the glass slide was covered with a clean glass slide to obtain a sandwich structure with a gap in the middle. Then, the hydrogel prepolymer was slowly injected into the gap of the sandwich structure. Finally, the sandwich structure was thermally polymerized for 20 min and soaked in 60% glycerol solution for swelling to obtain the photoelectric dual-mode sensor.

## Figures and Tables

**Table 1:** This work is compared with the existing relevant literature.

Cite	Δλ/nm	Electrical Signal	Stretchability	Water Retention Characteristics	Freeze Characteristics	Cycling Times
[37]	T: 45 nm S: 150 nm	×	1846.29%	√	√	T: 10 S: 10
[38]	T: 202 nm S: 154 nm	×	74.29%	√	√	T: 20 S: 30
[15]	T: × S: 195 nm	×	×	√	√	T: × S: 60
[39]	T: 10 nm S: ×	×	×	×	×	T: 20 S: ×
[40]	T: 56 nm S: ×	×	×	×	×	T: × S: ×
[41]	T: × S: 271 nm	√	50%	×	×	T: × S: 3000
[42]	T: × S: 198 nm	√	1050%	×	×	T: × S: 10^4^
[43]	T: × S: ×	×	×	×	×	T: 7 S: ×
[44]	T: × S: 127 nm	×	60%	×	×	T: × S: ×
This work	T: 48 nm S: 131 nm	√	253%	√	√	T: 20 S: 20

T: temperature; S: tensile strain; √: have relevant performance; ×: no relevant performance or mentioned.

## Data Availability

All data and materials are available on request from the corresponding author. The data are not publicly available due to ongoing research using a part of the data.

## Supplementary Materials

The following supporting information can be downloaded at: https://www.mdpi.com/article/10.3390/gels10080506/s1. Figure S1. The SEM of PMMA with (a) 170 nm, (b) 180 nm, (c) 205 nm, (d) 215 nm, (e) 222 nm, and (f) 227 nm. Figure S2. The SEM of PMMA photonic crystals with (a) 170 nm, (b) 180 nm, (c) 205 nm, (d) 215 nm, (e) 222 nm, and (f) 227 nm. Figure S3. The simulated results of PMMA photonic crystals with RSOFT software (a) 160 nm, (b) 180 nm, (c) 200 nm, (d) 220 nm, (e) 240 nm, and (f) 260 nm. Figure S4. The analysis of the relationship between PMMA diameter and reflection peak. Figure S5. The simulation results of PBG tuning of 235 nm PMMA PCs with lattice distance reduced to (a) 100.2%, (b) 93.267%, (c) 91.715%, (d) 62.959, (e) 48.07%, and (f) 38.279% of the initial lattice distance. Figure S6. The difference between reflected peaks of PMMA PCs and PMMA PCs sensors with different diameters. Figure S7. The SEM of the dual-mode photoelectric sensor with 235 nm PMMA PCs. Figure S8. The CIE picture of the dual-mode photoelectric sensor with 235 nm PMMA PCs with different tensile strain. Figure S9. The cycling reflective spectrum of photoelectric dual-mode sensor based on 235 nm PMMA PCs when the bending changed from 0° to 90°. Figure S10. The resistance of the photoelectric dual-mode sensor based on 235 nm PMMA PCs when the bending changed from 0° to 30°, 60°, and 90°. Figure S11 The resistance of the photoelectric dual-mode sensor based on 235 nm PMMA PCs when the bending changed from 0° to 30°, 60° and 90°.
