# Supplementary material for: Bio-Inspired Photoelectric Dual-Mode Sensor Based on Photonic Crystals for Human Motion Sensing and Monitoring
Source: Gels. 2024 Aug 1;10(8):506. doi: 10.3390/gels10080506 (PMC11353952; doi:10.3390/gels10080506)
Supplement: Supplementary file 1 [file gels-10-00506-s001.zip › gels-3079198-supplementary.pdf]

The Supporting Information for

Bio-Inspired Photoelectric Dual-mode Sensor Based on Photonic Crystals for Human Motion Sensing and Monitoring

Wenxiang Zheng [a], Zhibin Wang [a], Mengnan Zhang [a], Yanxin Niu [a], Yuchuan Wu [a], Pengxin Guo [a], Niu Zhang [b,*], Zihui Meng [a], Ghulam Murtaza [c,*], Lili Qiu [a,*]

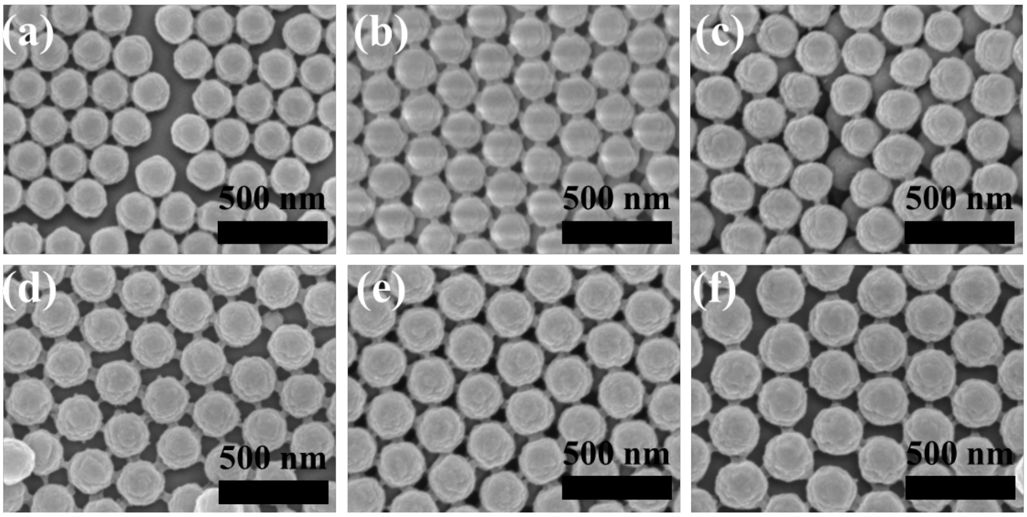

Figure S1 The SEM of PMMA with (a)170 nm, (b) 180 nm, (c) 205nm, (d) 215 nm, (e) 222 nm, (f) 227 nm.

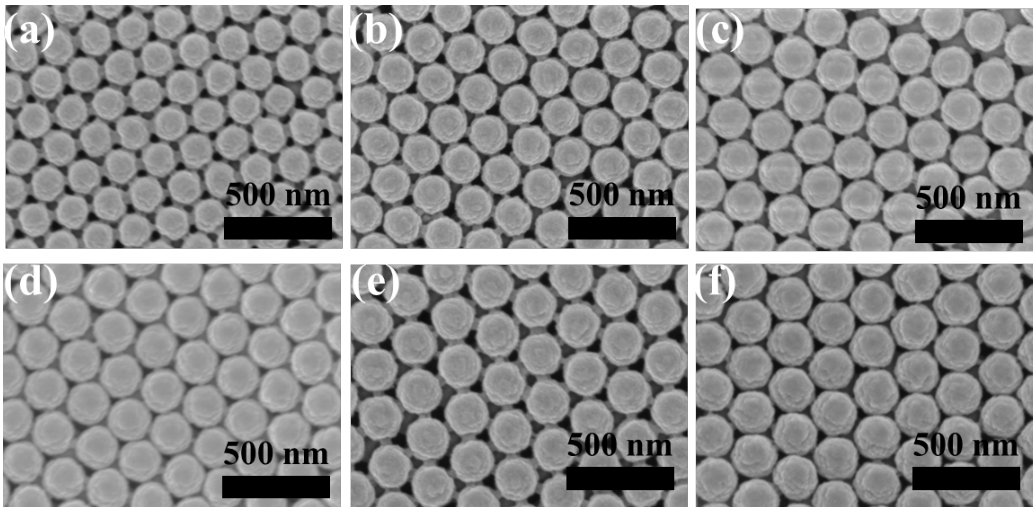

Figure S2 The SEM of PMMA photonic crystals with (a)170 nm, (b) 180 nm, (c) 205nm, (d) 215 nm, (e) 222 nm, (f) 227 nm.

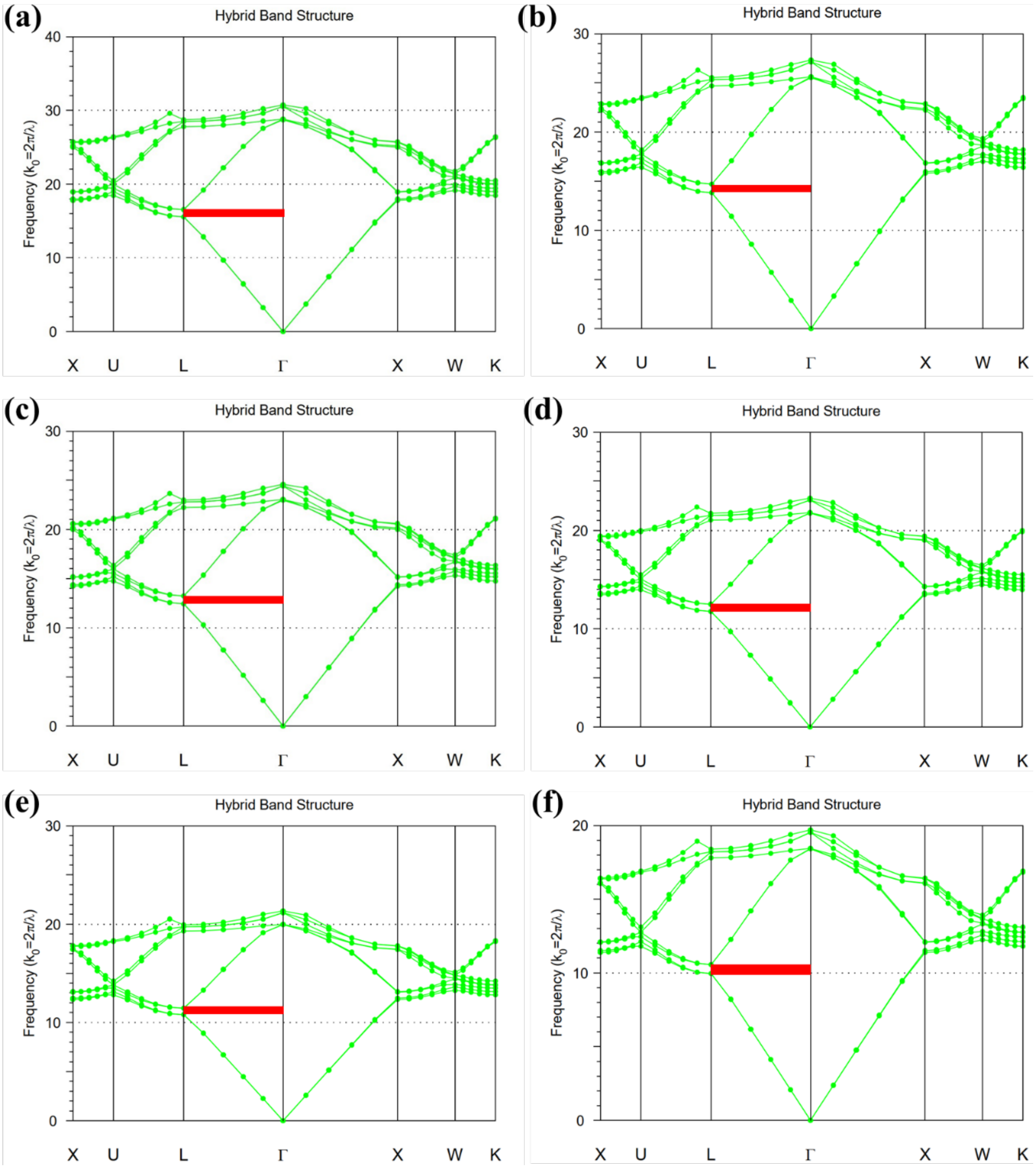

Figure S3 The simulated results of PMMA photonic crystals with RSOFT software (a) 160 nm, (b) 180 nm, (c) 200 nm, (d) 220 nm, (e) 240 nm, (f) 260 nm.

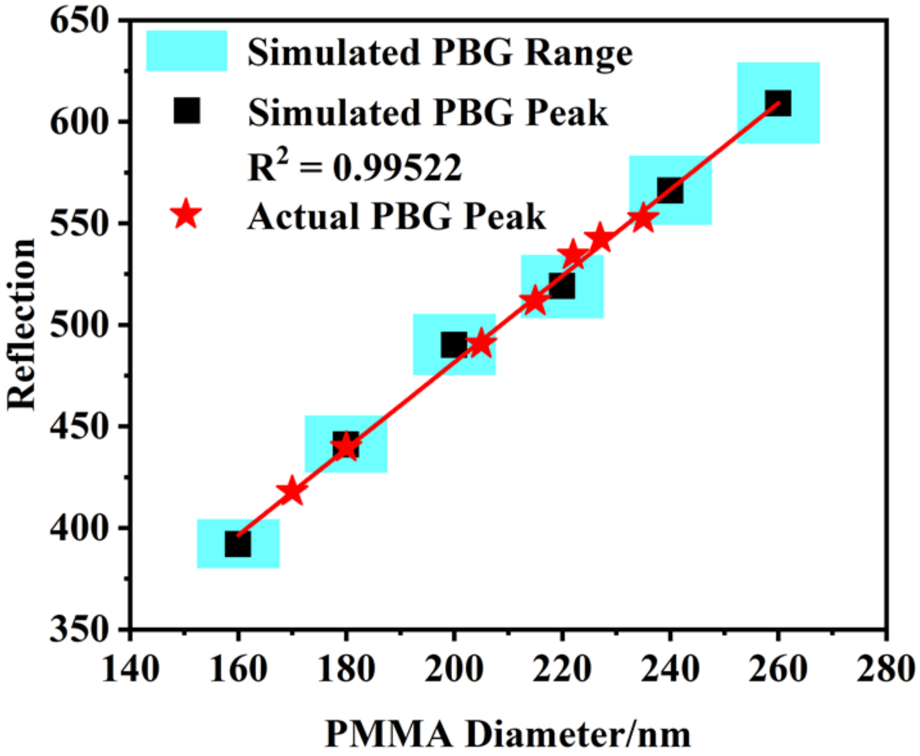

Figure S4 The analysis of the relationship between PMMA diameter and reflection peak.

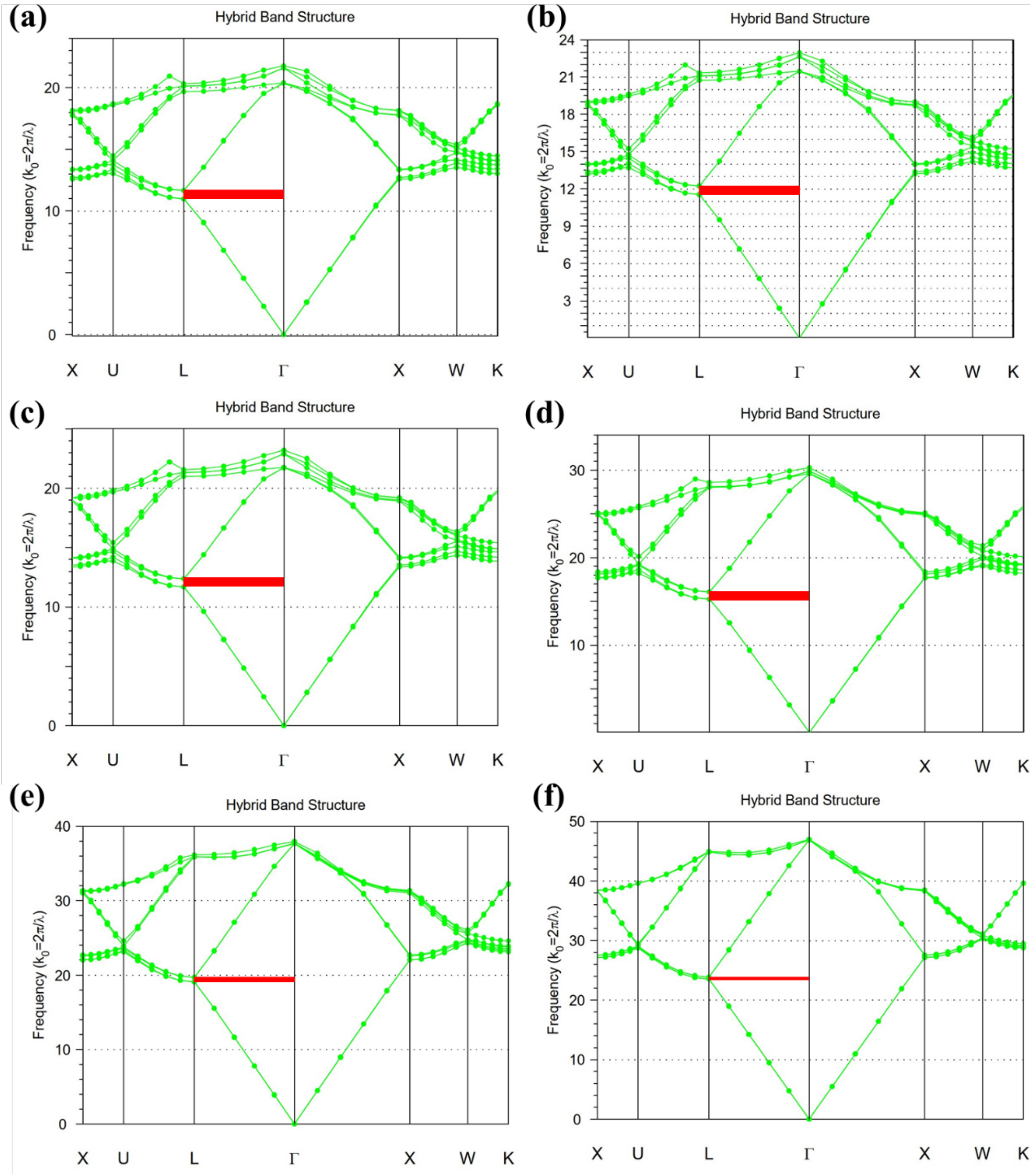

Figure S5 The simulation results of PBG tuning of 235 nm PMMA PCs with lattice distance reduced to (a) 100.2%, (b) 93.267%, (c) 91.715%, (d) 62.959, (e) 48.07% and (f) 38.279% of the initial lattice distance.

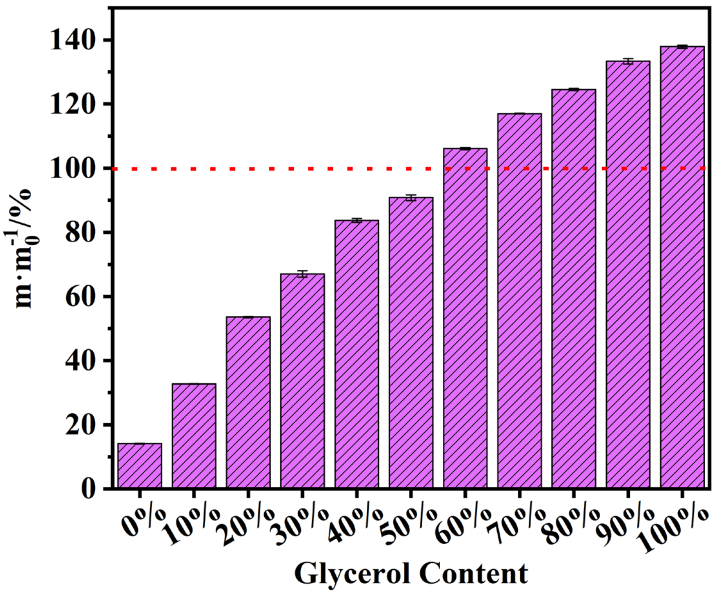

Figure S6 the water loss of 72h at 50% humidity and 35°C

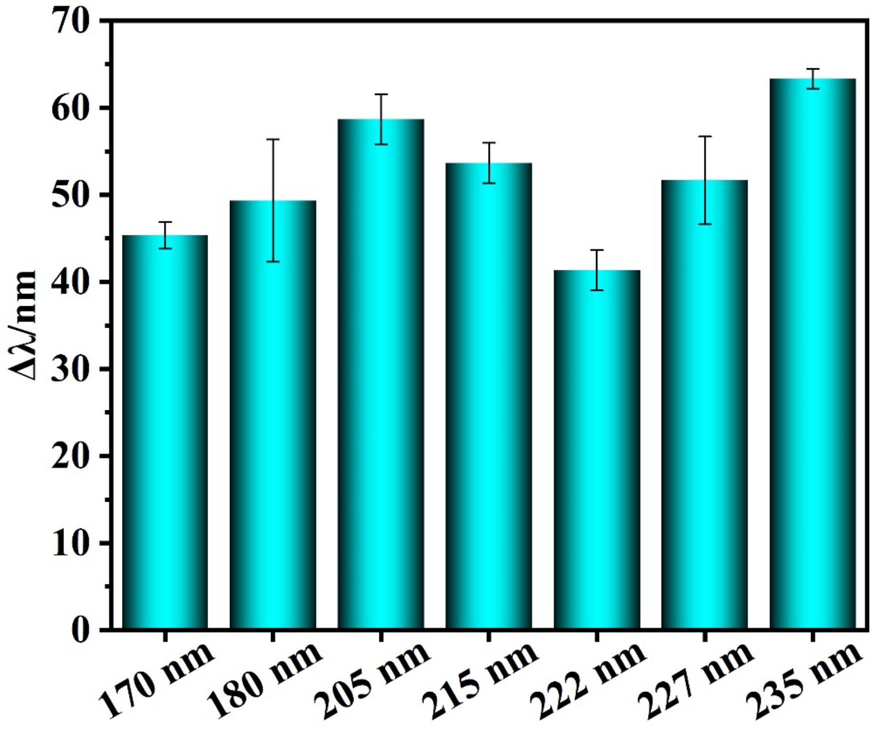

Figure S7 The difference between reflected peaks of PMMA PCs and PMMA PCs sensors with different diameters.

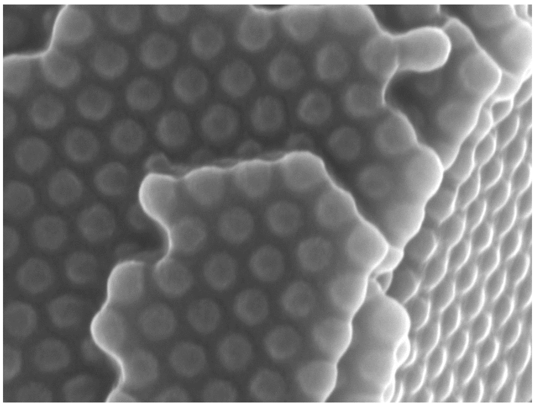

Figure S8 The SEM of the dual-mode photoelectric sensor with 235 nm PMMA PCs.

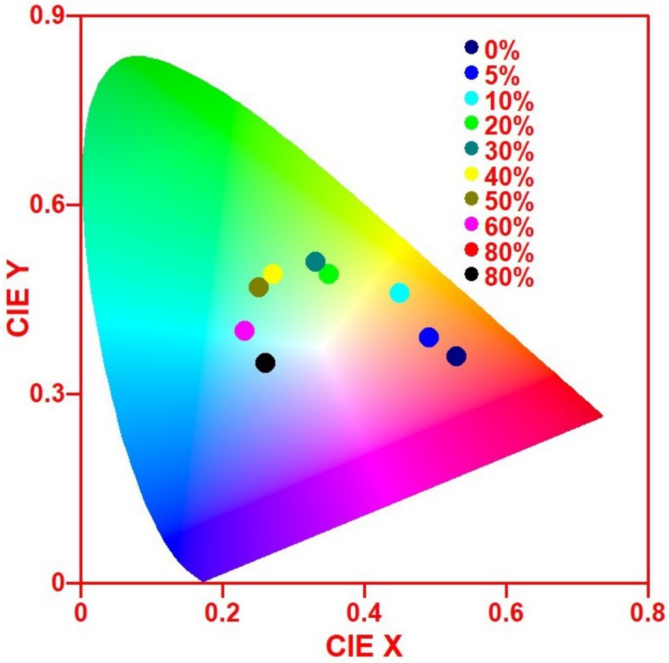

Figure S9 The CIE picture of the dual-mode photoelectric sensor with 235 nm PMMA PCs with different tensile strain.

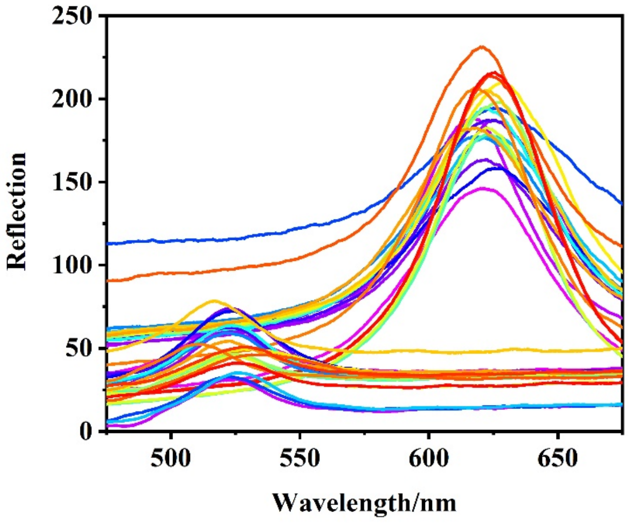

Figure S10 The cycling reflective spectrum of photoelectric dual-mode sensor based on 235 nm PMMA PCs when the bending changed from 0° to 90°.

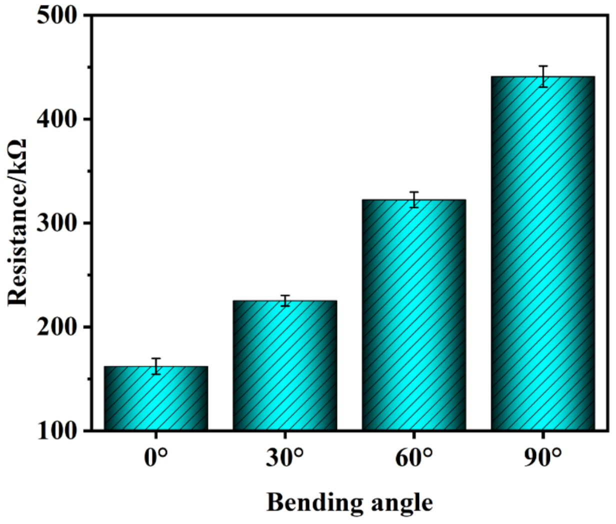

Figure S11 The resistance of the photoelectric dual-mode sensor based on 235 nm PMMA PCs when the bending changed from 0° to 30°, 60° and 90°.
